# Book Review: Seeking a Research-Ethics Covenant in the Social Sciences

**DOI:** 10.1177/15562646231200910

**Published:** 2023-09-10

**Authors:** Heidi Matisonn

**Affiliations:** The Ethics Lab, University of Cape Town & SARETI, University of KwaZulu Natal

While the view that ethical research practices are crucial for upholding scientific integrity, protecting human and animal welfare, and maintaining public trust in the research community is uncontroversial, there is some disagreement about the role of ethics committees/institutional review boards and formal regulations in ensuring that research is ethical. Van den Hoonaard has long been situated in the camp firmly opposed to the allegedly ‘brutal conditions caused by the capture of the research-ethics system by the medical model’ (pp.32–33). In this book, he sets out ‘to finally address the wrongs of ethics policies’ (p.xiv) by demonstrating what he persistently sees as ‘the failure of current ethics codes to serve as meaningful tools for researchers in the social sciences’ (p.2). He argues, in light of this, that ‘researchers in the social sciences should adopt the idea of a research-ethics covenant, a coherent model of ethics in research that can occupy a hallowed space in the hearts and minds of researchers’ (p.3). He describes a covenant as being a ‘solemn instrument of self-direction more suited to research in the social sciences’ (p.20) and proposes that such a covenant will ‘represent a series of tutorials where we all explore the ethical dimensions of social research’ (p.19). Such an approach, concludes van den Hoonaard, ‘may well be the only way to avoid the current ethics disorder that arises when researchers in the social sciences are mandated to adhere to the reigning biomedical framework and its various codes, policies, and ethics committees’ (p.105).

To convince us of the ‘colonization and capture’ (p. 10) of research ethics by the biomedical model, van den Hoonaard draws on experiences of social science researchers - anthropologists, sociologists, and historians - recounted to him personally or in published work. In chapter 2, he addresses concerns related to the audit culture of research institutions – specifically, that requisite pre-approval of studies fails to take account of the ‘emergent design’ (p.24) of (some) qualitative research.

Chapter 3 elucidates the views of some researchers who suggest that anonymity can preserve prevailing unequal power structures whereas avoiding it can ‘give power to the participants, allowing their words to be heard’ (p.39). Similarly, a concern with confidentiality is raised – because it can ‘protect secrecy’ it can ‘hinder transformative political action (p.40). Apropos informed consent, van den Hoonaard argues that the concept is deeply shaped by cultural diversity, disciplinary conventions, and fixed and fluid understandings, and sometimes depends ‘upon relationships with participants and the nature of the research discipline’. As such, ‘the habit of securing consent could simply amount to [what Corrigan 2003 refers to as] “empty ethics”.’ (p.40). Reporting that some social scientists are ‘particularly apprehensive about the extent to which vulnerability can become a catch-all, imaginary concept’ (p.40), van den Hoonaard takes aim at RECs/IRBs who, in ‘adhering’ to the ‘basic values of medical research’, tend to be ‘inconsistent in their approaches to evaluating social-science applications and ethnographic research’ (p.41) when it comes to their perceptions of what constitutes vulnerability.

Focused specifically on the discipline of anthropology and the work of anthropologists, chapter 4 maintains that the research approaches therein have been altered by the rise of ethics-review. Van den Hoonaard argues that ‘interviews are increasingly replacing ethnographic fieldwork’ largely because of the difficulties researchers have in obtaining ethics approval for the latter approach. Rather than a single code of ethics, which could pose ‘dangerous limitations’ (p.55), his favoured approach to meet the diverse needs of anthropologists is one that ‘empowers the individual anthropologist to make ethical decisions’ (p.56)

Chapter 5 entitled ‘Sociology and the New Ethics Disorder’ is written by Marco Marzano who argues that ‘neither in sociology nor in other social sciences (such as anthropology) was ethics regulation in any way bound up with any harm social research may have had on those involved in sociological research’ (p.57). In contrast, the ‘advent of the era of ethics regulation inflicted serious and considerable harm on the qualitative research of sociologists who immersed themselves professionally in the everyday lives of groups, communities, or organizations’ (p. 58). Marzano's attempt to defend covert research, rather controversially, invokes Machiavelli to justify the claim that ‘researchers who have told some small, innocent lies for the sake of the truth - that is, who have engaged in minor deception in order to be able get at a truth that they then make public - can be fully forgiven’ (p.68).

In the penultimate chapter, van den Hoonaard discusses several possible solutions to his concern: he describes the approach among critics of ethics-review which has the widest support - adapting the system to make it more ‘amenable for researchers in the social sciences’ (p.69). Such an approach includes ‘changing the deliberative and consultative process of research ethics committees, limiting the scope of these committees, or taking into account the social context of ethics review’ (p.70). Van den Hoonaard does not engage with this approach: absent from this book is reference to published work that helps social scientists work constructively and as equal partners within current ethics review guidance and systems or engagement with calls for social scientists to become members of research ethics committees to ensure that their particular methodologies are appropriately and competently reviewed and that these institutions are not dominated by positivists. Instead, he mentions the ‘admittedly small number who propose tightening research ethics codes and contrasts them with those who advocate for a ‘regulatory split between medical research and research in the social sciences’ (p.70). A fourth approach demands that policy makers eliminate all regulations, and it is in ‘that same breath’ (p.70) that van den Hoonaard advocates his covenantal approach which, because most of the book's content is dedicated to showing what is wrong with current ethics review processes, at least according to largely anecdotal reports from a few aggrieved social scientists, we only really learn about in the final chapter.

A covenantal approach to research ethics, we discover, calls for affirming an ethos of integrity in our professional lives (p.91). It is ultimately ‘about making ethics an integral part of the professional curriculum and/or training’ (p.92) and ‘ethical thinking and acting a fundamental part of all stages of our research and engagements’ (p.94). It is difficult to argue with calls to support ethics education and, given the proliferation of Offices of Research Integrity (or similar-named units) that have come into existence over the past few years, and the mandate of organisations like the Fogarty International Center of the National Institutes of Health to fund competitive applications for research ethics training initiatives, it seems few do. It thus seems curious for van den Hoonaard to claim that there is ‘a general lack of interest in ethics education’ (p.71).

Underneath all the frustration, at the heart of this book is an admirable idealism: that research misconduct arises from an absence of education rather than the presence of malfeasance. Van den Hoonaard's covenant is underpinned by the notion that ‘being an ethical researcher is not about exhibiting compliance with an external institutional machinery. It is about instilling in the researcher the sense that deeply ingrained ethical conduct is like being bound by a covenant - a sacred or hallowed trust if you will’ (p.101). Unfortunately, driven by his idealism, he seems to see ethics review as oppressive policing rather than as independent oversight, a position equivalent to arguing that if all people were raised correctly, we would not need laws and police. What the analysis overlooks is that harms to participants can and do occur due to the gap between moral cognition and moral action. Knowing what is right does indeed entail training and in this regard, van den Hoonaard is correct in emphasising the role of integrative ethics education for researchers. Doing what is right however, requires both regulation and enforcement, and a covenant, however sacred and well-intentioned, seems unlikely to be able to fulfil these roles.

